# POT1a and Components of CST Engage Telomerase and Regulate Its Activity in *Arabidopsis*

**DOI:** 10.1371/journal.pgen.1004738

**Published:** 2014-10-16

**Authors:** Kyle B. Renfrew, Xiangyu Song, Jung Ro Lee, Amit Arora, Dorothy E. Shippen

**Affiliations:** Department of Biochemistry and Biophysics, Texas A&M University, College Station, Texas, United States of America; Chinese Academy of Sciences, China

## Abstract

Protection of Telomeres 1 (POT1) is a conserved nucleic acid binding protein implicated in both telomere replication and chromosome end protection. We previously showed that *Arabidopsis thaliana* POT1a associates with the TER1 telomerase RNP, and is required for telomere length maintenance *in vivo*. Here we further dissect the function of POT1a and explore its interplay with the CST (CTC1/STN1/TEN1) telomere complex. Analysis of *pot1a* null mutants revealed that POT1a is not required for telomerase recruitment to telomeres, but is required for telomerase to maintain telomere tracts. We show that POT1a stimulates the synthesis of long telomere repeat arrays by telomerase, likely by enhancing repeat addition processivity. We demonstrate that POT1a binds STN1 and CTC1 *in vitro*, and further STN1 and CTC1, like POT1a, associate with enzymatically active telomerase *in vivo*. Unexpectedly, the *in vitro* interaction of STN1 with TEN1 and POT1a was mutually exclusive, indicating that POT1a and TEN1 may compete for the same binding site on STN1 *in vivo*. Finally, unlike CTC1 and STN1, TEN1 was not associated with active telomerase *in vivo*, consistent with our previous data showing that TEN1 negatively regulates telomerase enzyme activity. Altogether, our data support a two-state model in which POT1a promotes an extendable telomere state via contacts with the telomerase RNP as well as STN1 and CTC1, while TEN1 opposes these functions.

## Author Summary

Telomeres are required to stabilize the ends of linear chromosomes, and thus ensure genome integrity. Telomeric DNA is maintained though the action of both conventional and non-conventional DNA replication mechanisms. To ensure that chromosome ends are fully protected and fully replicated, telomeres dynamically oscillate between a closed (non-extendable) and an open (extendable) conformation throughout the cell cycle. The telomerase reverse transcriptase engages telomeres when they are in an extendable conformation. How this conversion occurs, how telomerase is recruited to the chromosome terminus and how telomerase action is terminated are unanswered questions. Here we provide evidence that POT1a, a telomerase accessory protein from the flowering plant *Arabidopsis*, helps to convert the telomere into a telomerase-extendable state through dynamic interactions with a critical telomere binding protein complex, and through stimulation of telomerase enzyme activity. The results of this study provide new insight into the regulation of telomeric DNA replication.

## Introduction

Eukaryotes face end-protection and end-replication problems due to the linear nature of their chromosomes and the limitations of conventional DNA replication. Telomerase averts these crises using its RNA subunit (TER) as a template to reiteratively synthesize G-rich repeat sequences on the 3′ single-strand extension (G-overhang) of the chromosome terminus. Both the single (ss) and double-strand (ds) portions of the telomere are host to protein complexes that modulate telomerase action and distinguish natural chromosome ends from double-strand breaks [1]–[4].

Telomeres vacillate between a telomerase extendable and a telomerase un-extendable state during the cell cycle [5], [6]. In G1, the G-overhang is sequestered, preventing the DNA terminus from eliciting a damage response, but also preventing telomerase access. In late S/G2 phase, telomerase is recruited to chromosome ends for DNA synthesis. Once telomerase extends the G-rich strand, the C-strand is replicated by DNA Polymerase α/primase [7], [8], followed by terminal DNA processing to create the 3′ G-overhang [9]. The terminus is then sequestered once again. These reactions are highly coordinated, and driven by the exchange of large replication/processing complexes on the G-overhang.

One telomere complex under intensive scrutiny is CST (Cdc13/CTC1, Stn1, Ten1), an RPA-like heterotrimer [10], [11] first identified in budding yeast. Cdc13 anchors CST to ss telomeric DNA via its central oligosaccharide-oligonucleotide binding domain (OB-fold) [12]. Genetic analysis of separation-of-function alleles reveals that Cdc13 maintains genome integrity and regulates telomere maintenance [13], [14]. Stn1 and Ten1 are also essential for telomere integrity, and their association with Cdc13 renders telomeres into an un-extendable state [15]–[17]. However, the CST heterotrimer is not static, and recent data show that Stn1 and Ten1 make contributions distinct from Cdc13 [18]. In addition, phosphorylation of Cdc13 in late S phase shifts the binding preference from Stn1 and Ten1 to the telomerase accessory factor Est1 [19], [20], converting the telomere into an extendable conformation. Est1 is a multifunctional protein that directly binds the TER subunit (Tlc1) as well as Cdc13. This interaction recruits telomerase to the chromosome end [21]–[24]. Consistent with its critical role in telomere maintenance, Est1 deletion causes progressive telomere shortening [25]. Est1 also stimulates the activity of telomerase on telomeric DNA [23], [26] likely through contacts with Cdc13 [27].

Mammalian telomeres are protected by an alternative complex termed shelterin. The six shelterin subunits include TRF1, TRF2, and RAP1, which are tethered to ds telomeric DNA and are bridged by TIN2 and TPP1 to the ss DNA binding protein POT1 [1], [28]. All shelterin components are critical for genome stability, and like budding yeast CST, may shift between sub-complexes during the cell cycle [29]. POT1 inhibits telomerase elongation *in vitro* by preventing substrate access [30], [31]. In contrast, the POT1-TPP1 heterodimer stimulates telomerase repeat addition processivity (RAP) by promoting substrate association and template translocation during telomerase extension [32]–[34]. In addition, TPP1 appears to directly contact the telomerase catalytic subunit TERT and thereby recruits telomerase to telomeres [35]–[37].

CST also exists in vertebrates and plants, although Cdc13 has been replaced by another large OB-fold containing protein, CTC1 [38]–[41]. In contrast to yeast where CST functions in both end protection and telomeric DNA replication [4], vertebrate CST primarily serves to promote telomere replication by stimulating C-strand fill-in and genome-wide replication rescue [42]–[45]. CTC1 and STN1 directly contact the telomerase activator proteins TPP1/POT1 [32], [46], [47]. Recent studies indicate that human CST negatively regulates telomerase by competing with TPP1/POT1 for telomeric DNA binding and by squelching the stimulation of telomerase RAP by TPP1/POT1 [46]. Thus, the interaction of TPP1/POT1 with CST is proposed to terminate G-strand synthesis by telomerase. While the molecular basis for the dynamic exchange between shelterin, telomerase and CST is unknown, shifting interactions between shelterin constituents [48], [49] prompted through posttranslational modification [20], [37], [50], [51] likely control telomere transactions.

*Arabidopsis* telomeres represent an intriguing blend of features from yeast and vertebrates. Only a subset of shelterin components can be discerned in plants, and although the *Arabidopsis* CST complex is structurally analogous to mammalian CST, it appears to play a role in chromosome end protection. Loss of any of the *Arabidopsis* CST subunits elicit dramatic telomere shortening, increased ss telomeric DNA, and chromosomal fusions [38], [39], [41], culminating in stem cell failure [52]. Notably, TEN1 is detected at a significantly smaller fraction of telomeres than CTC1 [39], [41]. In addition, unlike plants lacking STN1 or CTC1, *ten1* mutants have higher levels of telomerase enzyme activity overall, and generate longer telomere repeat arrays *in vitro*, indicating that TEN1 negatively regulates telomerase activity [41].

*Arabidopsis* harbors two TER genes encoding RNAs that assemble into different RNP complexes with opposing functions. TER1 is a canonical TER subunit required for telomere maintenance, whereas TER2 negatively regulates telomere synthesis by the TER1 RNP in response to DNA damage [53], [54]. *Arabidopsis* encodes several telomerase accessory factors, but notably the two Est1-like proteins play no obvious role in telomere maintenance and rather are implicated in the regulation of the meiotic cell cycle [55]. POT1a, one of three *A. thaliana* POT1 paralogs [56]–[58] exhibits properties reminiscent of Est1. POT1a associates with TER1, and localizes to telomeres in S phase [59]. Moreover, plants lacking POT1a are defective in telomere maintenance, and undergo progressive telomere shortening. In addition, *pot1a* mutants have reduced telomerase activity *in vitro* [59]. These findings indicate that POT1a positively regulates telomerase enzyme activity and promotes telomere repeat synthesis on chromosome ends.

In this study, we further explore the role of POT1a. We report that POT1a is not required to recruit telomerase to telomeres, but is required for telomerase to maintain telomere tracts. Our biochemical data indicate that POT1a stimulates telomerase enzyme activity, likely by enhancing its RAP. We further show that POT1a directly contacts STN1 and CTC1 *in vitro*, and its association with STN1 is mutually exclusive of TEN1-STN1 binding. Finally, we demonstrate that CTC1 and STN1, but not TEN1, interact with enzymatically active telomerase *in vivo*. These findings suggest a model in which POT1a promotes telomere maintenance by activating telomerase at chromosome ends. The data further suggest that the opposing functions of POT1a and TEN1 in telomerase regulation may contribute to the switch from telomerase extendable to the telomerase un-extendable state.

## Results

### POT1a is not required for TERT association with chromosome ends

**Figure 1:**
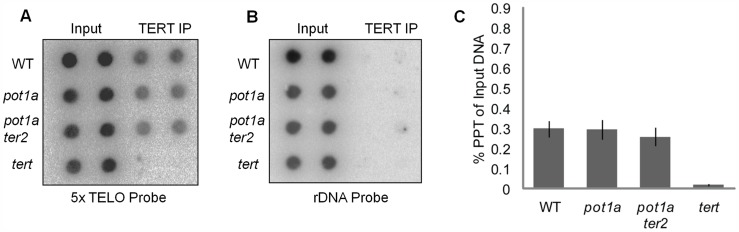
Telomerase associates with telomeres in the absence of POT1a. (A) Results of ChIP assays using TERT antibody in wild type, *pot1a, pot1a ter2*, and *tert* seedlings. Signal was assessed by dot blot using a telomeric probe. Reactions shown are technical duplicates. (B) Membrane was stripped and re-hybridized with a rDNA oligonucleotide probe. (C) Quantification of TERT ChIP. IP signal is represented as percent precipitation of input DNA. Error bars represent standard error of the mean from three independent biological replicates.

Chromatin immunoprecipitation (ChIP) was used to investigate whether POT1a is needed for telomerase association with telomeres. As expected, the telomerase catalytic subunit TERT [60] could be detected at telomeres in rapidly dividing young wild type seedlings (Fig. 1A). However, there was no significant difference in the level of telomere-bound TERT in *pot1a* mutants versus wild type (Fig. 1A and C). One possible explanation is that the TERT signal includes telomere-bound TER2 RNP. Since POT1a does not interact with TER2 [54], loss of this protein is not expected to perturb the alternative telomerase RNP. To address this possibility, we generated plants doubly deficient in POT1a and TER2. ChIP assays performed on *pot1a ter2* mutants showed the same level of telomere-bound TERT as in wild type plants (Fig. 1A and C). We conclude POT1a is not required for TERT recruitment to telomeres.

### POT1a stimulates activity of the TER1 telomerase RNP

If POT1a is not required for telomerase's association with chromosome ends, how does it promote telomere maintenance? One possibility is that POT1a directly modulates telomerase enzyme activity. The conventional telomere repeat amplification protocol (TRAP) assay shows an ∼13 fold decrease in telomerase activity in *pot1a* relative to wild type extracts [59]. This change in enzyme activity is not due to altered expression of TERT and TER1 transcripts or genes previously shown to inhibit telomerase activity such as TER2 and TEN1 (Fig. S1). Attempts to develop a direct primer extension assay in *Arabidopsis* have been unsuccessful thus far. To obtain a more accurate gauge of the distribution and quantity of the products of *Arabidopsis* telomerase, we used a modified version of the TRAP assay, telomerase processivity TRAP (TP-TRAP), developed to provide an indication of mammalian telomerase RAP [41], [61]. Pilot reactions with an oligonucleotide bearing five telomere repeats yielded a discrete band of the expected size (Fig. S2), indicating that the PCR amplification step of TP-TRAP gives a reliable assessment of the length of a telomere repeat array generated in the PCR reaction.

**Figure 2:**
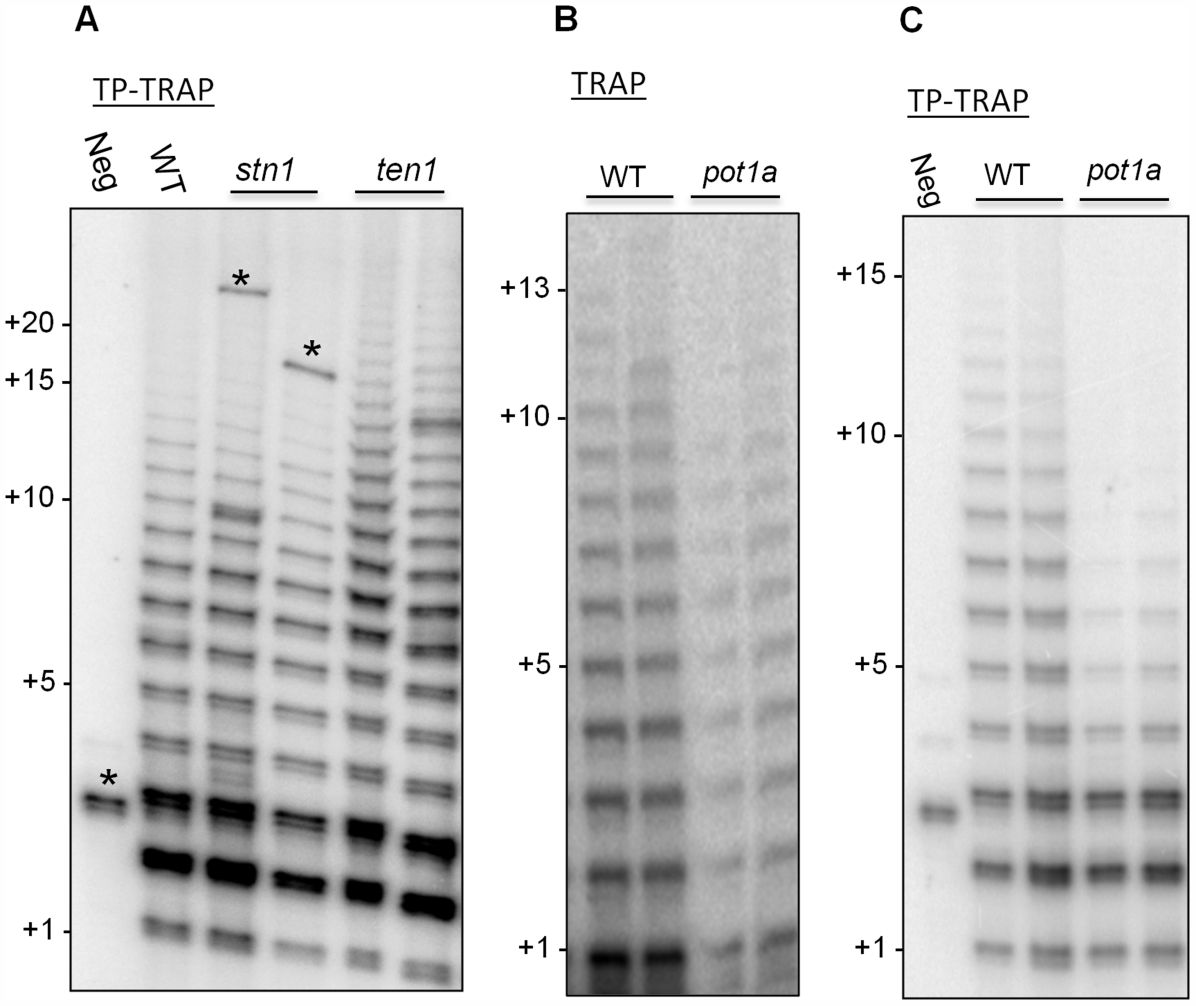
POT1a promotes synthesis of long telomere repeat arrays. (A) TP-TRAP assay results performed on flower extracts from wild type, and two independent *stn1*, and *ten1* mutants. Negative control is without extract to monitor PCR contamination. Asterisks indicate non-specific amplification products. (B) Conventional TRAP results from wild type and *pot1a* seedling extracts. Results from two independent seedling extractions are shown. (C) TP-TRAP results for *pot1a* and wild type seedling extracts. Results from two independent seedling extracts are shown. Negative control is without extract.

TP-TRAP performed with wild type *Arabidopsis* extract generated a broad distribution of elongation products, including high molecular weight species corresponding to the addition of at least 15 TTTAGGG repeats (Fig. 2A and C). As expected, extract from *ten1* mutants, but not *stn1* or *ctc1* mutants, generated slightly longer products than wild type (Fig. 2A and S3) [41], supporting the conclusion TEN1 negatively regulates telomerase activity and further that this is a unique property of this CST subunit. The TP-TRAP results for *pot1a* mutants were markedly different and showed a dramatic reduction in high molecular weight products relative to wild type (Fig. 2C). While standard TRAP assays show a general decrease in telomerase activity in *pot1a* mutants (Fig. 2B), the TP-TRAP indicated that the defect lies in the production of long arrays of telomere repeats (Fig. 2C). The primer is in vast excess over telomerase in TP-TRAP reactions as in conventional TRAP and the direct primer extension assay. Consequently, long products are unlikely to be generated by telomerase dissociation and rebinding the same primer molecule. The data are consistent with the notion that POT1a stimulates RAP.

**Figure 3:**
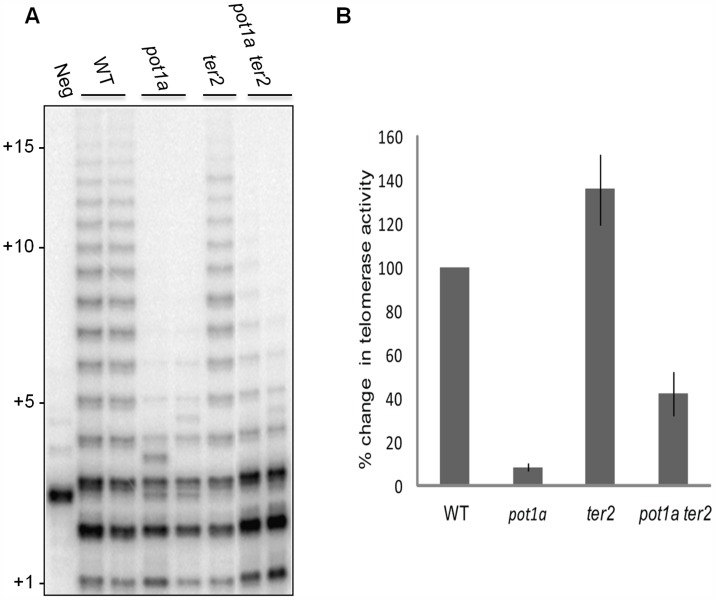
POT1a stimulates telomerase activity of the TER1 RNP. (A) TP-TRAP analysis from two independent biological replicates wild type, *pot1a, ter2*, and *pot1a ter2* mutants. (B) Results of quantitative TRAP (qTRAP). Error bars represent standard error of the mean from three biological replicates.

To determine if the decreased telomerase activity associated with *pot1a* mutants is specific to the TER1 RNP complex, we performed TP-TRAP on *ter2* seedling extracts. The product profiles were nearly identical to wild type (Fig. 3A), indicating the TER1 RNP efficiently synthesizes telomeric DNA in wild type plants. We confirmed that POT1a modulates the TER1 RNP by analyzing *pot1a ter2* mutants. Long products were reduced in the double mutants, but not to the same extent as *pot1a* (Fig. 3A). In agreement with previous results showing that TER2 negatively regulates TER1 RNP [54], quantitative TRAP (qTRAP) revealed a higher level of telomerase activity in *ter2* mutants relative to wild type (Fig. 3B), which could explain why the TP-TRAP and qTRAP signal is higher in *pot1a ter2* than *pot1a* (Fig. 3A and B). Since the TER1 RNP is the only functional telomerase complex in *pot1a ter2* mutants, the data indicate POT1a distinctly modulates this complex.

### Telomere dysfunction is exacerbated in plants lacking telomerase and STN1 or CTC1

**Figure 4:**
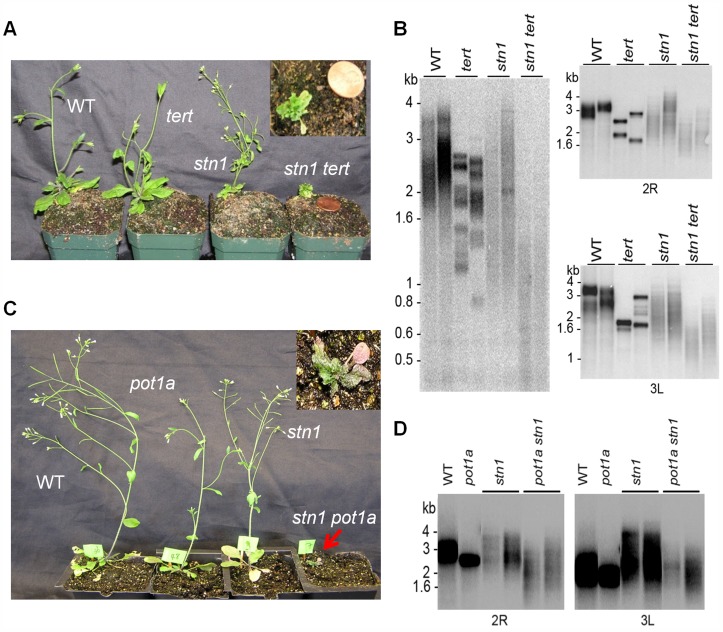
POT1a acts with telomerase to partially rescue the telomere dysfunction of *stn1* mutants. (A) Morphology of wild type, *stn1, tert*, and *stn1 tert* double mutants. Telomere length analysis assessed by TRF (B, left panel) and PETRA (B, right panel; D) for the genotypes indicated. In each case, results for two independent plants are shown. For PETRA, telomeres on the right arm of chromosome 2 (2R) or the left arm of chromosome 3 (3L) were analyzed. Wild type controls were segregated from either the *stn1* × *tert* cross (B) or the *stn1* × *pot1a* cross (D). (C) Morphology of wild type, *stn1, pot1a*, and *stn1 pot1a* double mutants.

In both yeast and vertebrates, CST plays a key role in controlling G-overhang access to telomerase and DNA Pol-α [4], [7], [46]. To test whether telomerase acts in concert with CST for telomere maintenance, we used a genetic approach. As expected, *ctc1* and *stn1* mutants exhibited severe morphological aberrancies including irregular phyllotaxy, fasciated stems, and reduced fertility (Fig. 4A and C, and S4A; [38], [39]). These phenotypes were even more pronounced when telomerase was inactivated in *stn1* and *ctc1* plants (Fig. 4A and S4A). Progeny lacking CTC1/STN1 and TERT were rarely recovered, and when they were, double mutants arrested in a dwarf vegetative state without producing germline tissue (Fig. 4A and Fig. S4A). Telomere length was examined using Terminal Restriction Fragment (TRF) analysis or Primer Extension Telomere Repeat Amplification (PETRA) when insufficient material was available for TRF. Consistent with previous studies [38], [39], *stn1* or *ctc1* mutants displayed shorter, more heterogeneous telomere tracts than wild type plants. In contrast, while telomeres in *tert* mutants consisted of a discrete, homogeneous population of bands shorter than wild type (Fig. 4B and Fig. S4B) [62]. The telomeres of plants lacking either CTC1 or STN1 and telomerase were dramatically shorter with some telomeres dipping below the critical threshold of 1 kb (Fig. 4B and Fig. S4B), which triggers telomere fusions [63]. We conclude telomerase is capable of extending telomeres devoid of CTC1 or STN1 to partially alleviate their dysfunction. However, given the very severe telomere deprotection phenotype associated with the loss of CST, these epistasis experiments do not rule out the possibility that STN1 or CTC1 engage telomerase and modulate its activity *in vivo*.

To determine if POT1a is required for telomerase to mitigate telomere defects in STN1/CTC1 deficient plants, we evaluated *pot1a ctc1* and *pot1a stn1* double mutants. We were unable to recover viable *pot1a ctc1* mutants. However, *stn1 pot1a* mutants exhibited similar morphological defects as *stn1 tert* plants (Fig. 4C). In addition, molecular analysis revealed the same type of telomere aberrations (Fig. 4D). Thus, the absence of POT1a renders *stn1* mutants incapable of employing telomerase as a recovery mechanism (Fig. 4B). These findings support the conclusion that POT1a is required to activate telomerase at chromosome ends.

### POT1a associates with CTC1 and STN1, but not TEN1 *in vitro*

**Figure 5:**
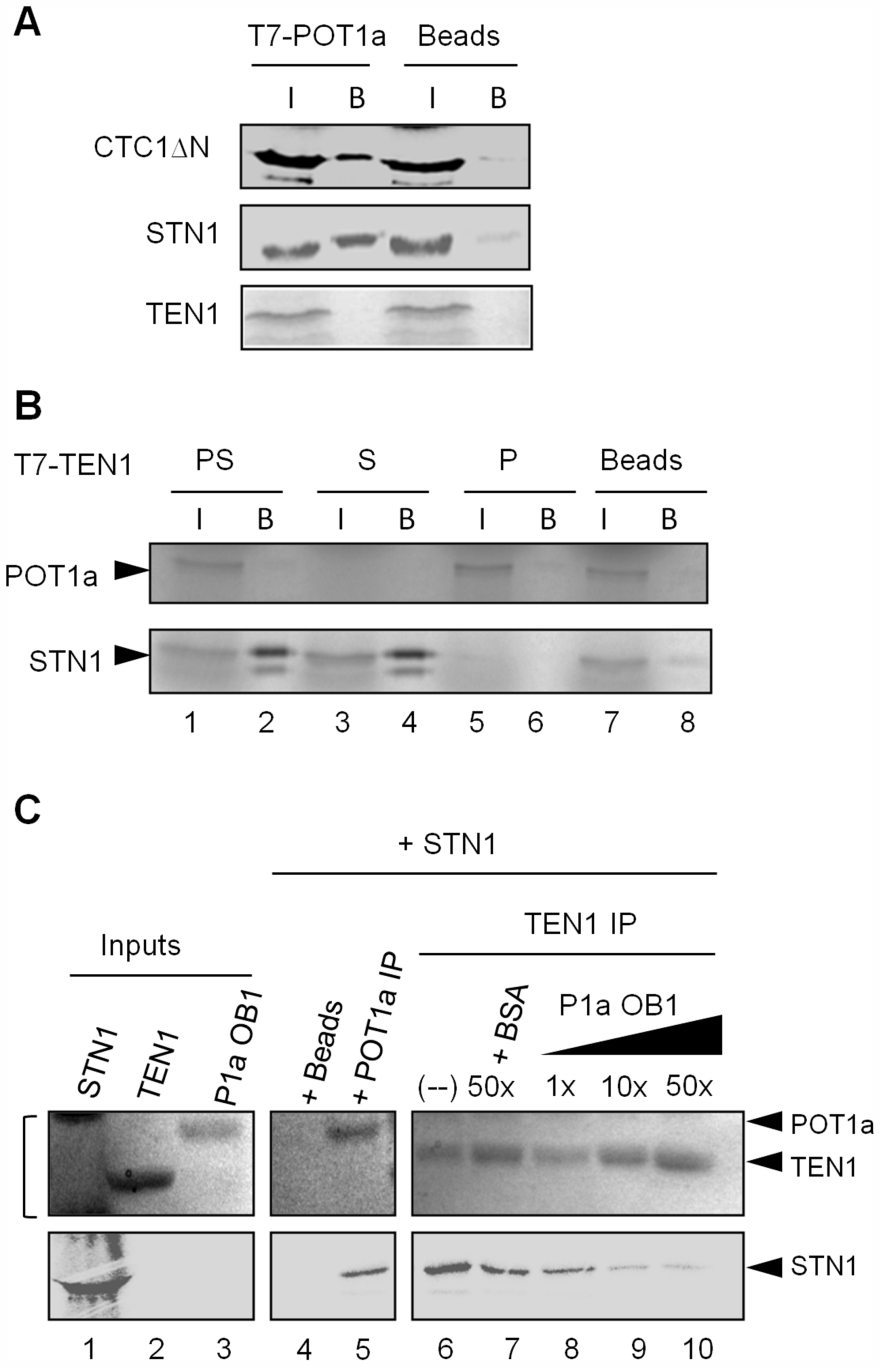
POT1a associates with CTC1 and STN1 *in vitro*. (A) *In vitro* co-immunoprecipitation (co-IP) results for RRL-expressed T7-tagged POT1a interactions with labeled CTC1ΔN, STN1, and TEN1. Negative control (beads conjugated with T7-tag antibody) was performed without tagged POT1a. (I) denotes protein input, (B) indicates bound protein. (B) Co-IP results for RRL-expressed T7 tagged TEN1 with labeled POT1a (P; lane 6), STN1 (S; lane 4) or both proteins (“PS”, lane 2). The beads control contained no T7 tagged TEN1 (lane 8). (C) *In vitro* Co-IP competition assay using *E. coli*-expressed TEN1 and POT1a OB1 detected by coomassie stain, and RRL-expressed ^35^S methionine labeled STN1 detected by autoradiography. Protein inputs are shown in lanes 1–3. Bracket adjacent to lane 1 denotes non-specific RRL proteins in the STN1 expression reaction (lane 1, top). TEN1 was incubated with STN1 and increasing concentrations of POT1a OB1 (lanes 8–10). 50× BSA was used as a control (lane 7) IP of POT1a was performed independently to verify its interaction with STN1 (lane 5). Beads alone was used to monitor background binding of STN1 protein (lane 4).

Recent studies show that human POT1 and mouse POT1b bind CTC1 and STN1 [46], [47], [64]. Additional contacts between TPP1 and CTC1 and TPP1 and STN1 have been observed [46], [64], [65]. Therefore, we asked if POT1a binds individual CST subunits *in vitro* via co-immunoprecipitation assays using rabbit reticulocyte lysate (RRL) expressed proteins. We were unable to express intact full length CTC1, and so we employed an amino-terminal deletion construct (CTC1ΔN) that was sufficient to bind STN1 and the DNA Pol α subunit, ICU2 [4], [39]. POT1a was tagged with T7 on its amino terminus and immunoprecipitation (IP) was performed using T7 antibody-conjugated agarose beads. Binding was assessed by the ability of POT1a to co-precipitate ^35^S-methionine labeled CTC1ΔN, STN1, or TEN1. We detected POT1a binding to CTC1ΔN and STN1, but no interaction between TEN1 and POT1a was observed (Fig. 5A).

Since TEN1 and STN1 form a heterodimer, we considered the possibility that POT1a might compete with TEN1 for STN1 binding. We first tested if STN1 can simultaneously bind POT1a and TEN1. TEN1 was T7 tagged, and incubated with labeled STN1 (Fig. 5B, lane 4), POT1a (Fig. 5B, lane 6) or both proteins (Fig. 5B, lane 2) followed by IP. In the reaction containing STN1 and POT1a, only STN1 was detected in the TEN1 IP (Fig. 5B, lane 2). Because TEN1 does not bind POT1a (Fig. 5A and Fig. 5B, lane 6), this result argues that STN1 binding to TEN1 and POT1a is mutually exclusive.

Next, we asked whether POT1a can compete with TEN1 for STN1 binding *in vitro*. We expressed and purified *E. coli* TEN1 protein as well as the first OB-fold of POT1a (POT1a OB1), which is sufficient for POT1a-STN1 interaction *in vitro* (Fig. S5 and Fig. 5C, lane 5). A competition assay was performed by incubating TEN1 with RRL-expressed [35S]-methionine labeled STN1 in the presence of increasing amounts of POT1a OB1. Following TEN1 IP, *E. coli*-expressed proteins (TEN1 and POT1a OB1) were monitored by coomassie stain (Fig. 5C *top*) and STN1 by autoradiography (Fig. 5C *bottom*). As expected, TEN1 pulled down STN1 (Fig 5C, lane 6). At an equal molar ratio of POT1a OB1 to TEN1, the TEN1-STN1 interaction persisted (Fig. 5C, lane 8). However, a ten-fold excess of POT1a OB1 significantly reduced STN1 in the TEN1 IP (Fig. 5C, lane 9). In contrast, 50-fold excess bovine serum albumin did not dislodge STN1 from TEN1 (Fig. 5C lane 7). Because *E. coli* POT1a OB1 directly binds STN1 (Fig. 5C, lane 5), these data support the conclusion that STN1 binding to POT1a and TEN1 is mutually exclusive. However, because excess POT1a OB1 is required to disrupt the STN1-TEN1 interaction, the data indicate that STN1 has a higher affinity for TEN1 than POT1a OB1.

### STN1 and CTC1, but not TEN1, associate with enzymatically active telomerase *in vivo*

**Figure 6:**
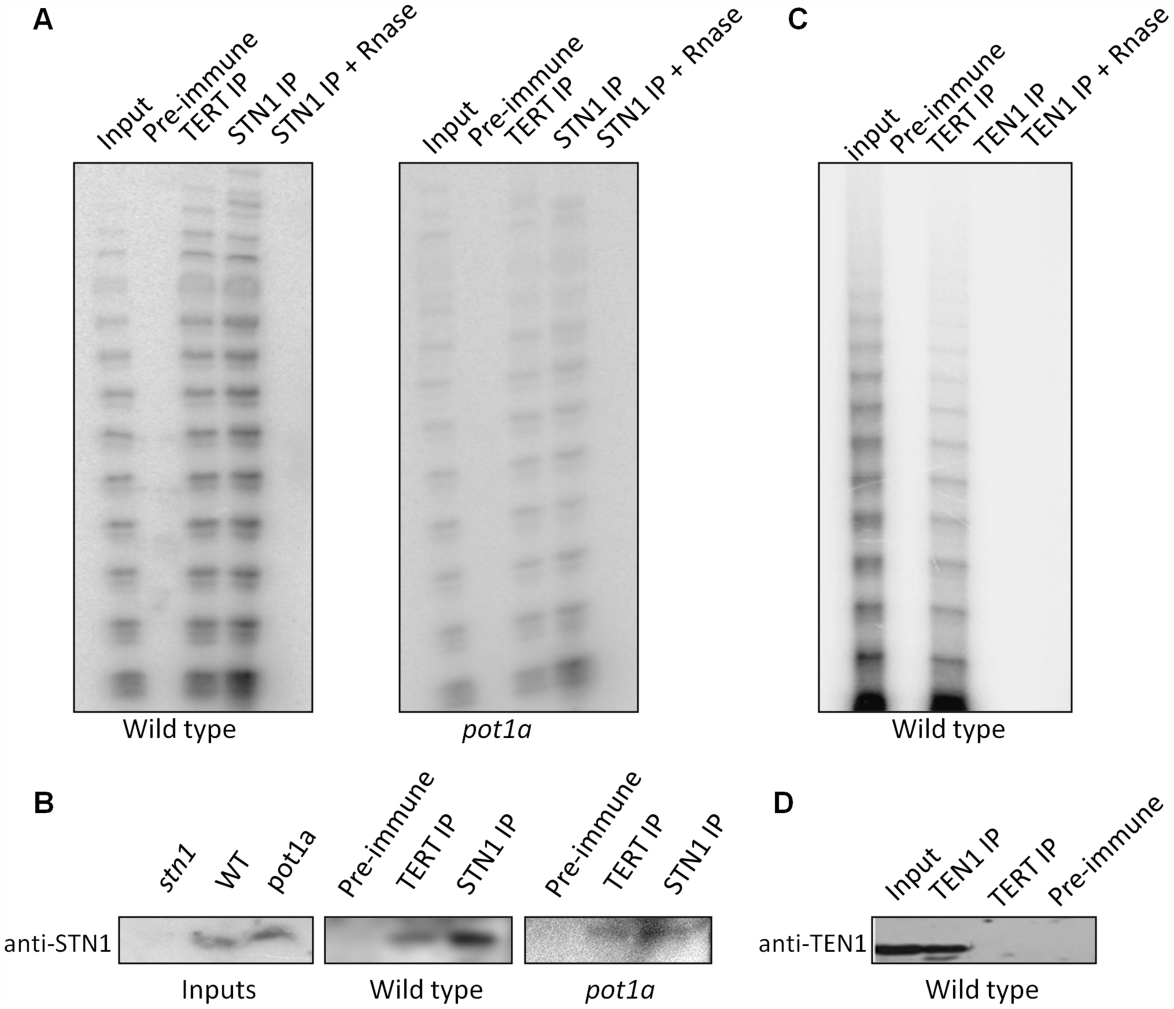
STN1, but not TEN1 associates with enzymatically active telomerase *in vivo*. (A) Protein extract from wild type or *pot1a* seedlings was used for immunoprecipitation with STN1 or TERT antibody. IP samples and extract input were subjected to conventional TRAP (A) or western blot (B) analysis with STN1 antibody. (C) Protein extract from wild type seedlings was used for IP with TEN1 antibody. IP samples and extract input were subjected to conventional TRAP or (D) western blot to monitor for TEN1 protein after IP.

The discovery of *in vitro* interactions between POT1a with STN1 and CTC1 raised the possibility that these CST components associate with enzymatically active telomerase *in vivo* (Fig. 6). To test this idea, we generated a STN1 antibody that could be used for IP-TRAP. Western blot analysis confirmed that the antibody specifically recognizes STN1 (Fig. 6B). IP-TRAP using TERT antibody as a control revealed abundant telomerase activity (Fig. 6A). Strikingly, IP-TRAP with STN1 antibody gave a similar result. Western blot analysis verified that STN1 was precipitated in the reaction (Fig. 6B). Telomerase activity was not detected in an IP with pre-immune sera and was removed by RNaseA treatment, indicating that the STN1 interaction with telomerase was specific. Importantly, STN1 protein was present in the TERT IP (Fig. 6B), confirming the association of these molecules *in vivo*. IP of a transgenic CTC1-CFP protein also pulled down active telomerase as well as POT1a (Fig. S6). These findings indicate that both STN1 and CTC1 are associated with enzymatically active telomerase *in vivo*.

We asked if POT1a was essential for the STN1-telomerase interaction by repeating the STN1 IP-TRAP experiment in a *pot1a* mutant. Telomerase activity and TERT were detected in the STN1 IP of *pot1a* extracts (Fig. 6A and B). As expected, telomerase activity was visibly decreased in this background ([59]; Fig. 6B). These data indicate that telomerase can associate with STN1 in the absence of POT1a. The data also support the conclusion that POT1a is not necessary for telomerase localization to telomeres, but is required to promote the full activation of telomerase.

Finally, we performed IP-TRAP with our TEN1 antibody to test if TEN1 is associated with active telomerase. In marked contrast to STN1 and CTC1, telomerase activity was not observed in the TEN1 pull down (Fig. 6C). Moreover, TEN1 protein could not be detected in the TERT IP (Fig. 6D). We conclude that TEN1 is not associated with enzymatically active telomerase *in vivo*, consistent with its role as a negative regulator of telomerase activity.

## Discussion

Telomere accessibility to telomerase is tightly regulated during the cell cycle. Whereas aspects of telomerase recruitment are similar in yeast and vertebrates, many questions remain unanswered, in part because the specific proteins that mediate these interactions are not well conserved [29]. In this study, we investigated how the interplay between POT1a and CST in *Arabidopsis* promotes telomere maintenance. Like the budding yeast recruitment factor Est1 [21], [22], [25], [27], POT1a directly contacts the canonical TER, TER1 [53], and is required for robust telomerase activity *in vitro* and telomere maintenance *in vivo* [59]. However, unlike Est1 [66], we found that POT1a is not necessary for the telomere localization of TERT. The TERT interaction with telomeres was also unperturbed in plants doubly deficient in POT1a and TER2, indicating TERT is not tethered to telomeres through the TER2 RNP. How telomerase is recruited to chromosome ends in the absence of POT1a is unclear. In yeast, Ku provides an alternative route for telomerase recruitment in G1 [66]. However, Ku inhibits telomere synthesis in plants [67], [68], and thus this mechanism is not used to dock telomerase at *Arabidopsis* telomeres. The TRF-like protein AtTRB1 was recently shown to interact with telomeres and to contact TERT, suggesting that it might be involved in telomerase recruitment [69]. Another potential telomerase recruitment factor is HOT1, which stimulates telomerase recruitment in mammals through contacts with telomeric DNA and the telomerase RNP independent of shelterin [70]. Notably, *Arabidopsis* has a putative HOT1 ortholog, but lacks several of the core shelterin components, including TPP1, which is implicated in recruiting vertebrate telomerase [33], [36].

Although POT1a is not required for telomerase recruitment, it is required for the enzyme to extend telomere tracts *in vivo* ([59]; this study). Our data indicate POT1a directly stimulates telomerase catalysis. Using a modified version of the TRAP assay to gauge the length of telomerase products, we discovered that POT1a is necessary for the synthesis of long telomere repeat arrays. An attractive model is that POT1a promotes telomerase RAP, as shown for other telomerase-associated OB-fold bearing proteins such as human TPP1 and *Tetrahymena* Teb1 [32], [35], [71]. However, in the absence of a direct primer extension assay for *Arabidopsis* telomerase, we cannot exclude the possibility that POT1a affects some other parameter of telomerase enzymology (e.g. nucleotide addition processivity, nucleotide binding affinity or affinity for the DNA primer).

Once telomerase binds the telomere, how is its activity controlled? CST has a central role to play in this regard, but precisely how it interfaces with telomerase and whether this association stimulates or represses telomerase differs in yeast and vertebrates. Our analysis indicates that CST is not required to recruit *Arabidopsis* telomerase to chromosome ends. We found that telomerase can act on telomeres lacking CTC1 or STN1, partially alleviating the telomere dysfunction and the aberrant morphological defects associated with these mutations. Importantly, telomere extension in CTC1 and STN1 deficient plants is dependent upon POT1a, supporting the conclusion that POT1a is required to promote telomere maintenance.

In mammals, CST interaction with POT1 orthologs is linked to telomerase termination [46] and G-overhang maturation [47]. In contrast, we find that STN1 and CTC1 like POT1a are associated with enzymatically active telomerase in *Arabidopsis* [59]. Our experiments do not distinguish whether these telomerase interactions occur on or off the telomere. Nevertheless, since CTC1 can be detected at *Arabidopsis* telomeres even in cells arrested in G1 (Surovtseva et al 2009), we postulate that telomerase associates with CTC1 and STN1 on the G-overhang during S phase to facilitate telomere repeat incorporation (see below).

We found a direct interaction between POT1a with both STN1 and CTC1, but not TEN1 *in vitro*. Our data indicate that STN1 interaction with POT1a and TEN1 is mutually exclusive. FurthermoreTEN1 unlike STN1 and POT1a is not associated with active telomerase *in vivo*. These observations are consistent with a role for TEN1 in negative regulation of telomerase enzyme activity [41]. Intriguingly, TEN1 may only transiently associate with *Arabidopsis* telomeres. CTC1 can be detected at ∼50% of the *Arabidopsis* chromosome ends [39]. Since only half of the *Arabidopsis* telomeres carry G-overhangs [72], essentially all of the G-overhangs are bound by CTC1. In contrast, TEN1 can only be detected at 11% of the telomeres [41], implying that it dynamically binds telomeres and does not function exclusively in the context of a trimeric CST complex.

**Figure 7:**
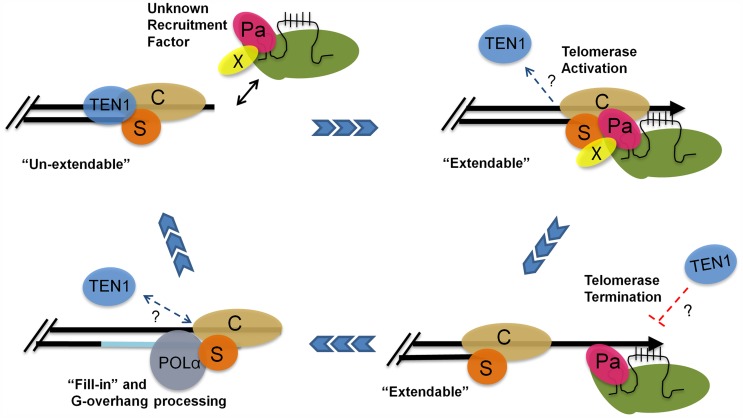
A model for telomere replication in *Arabidopsis*. In the un-extendable state, telomeres are bound by the heterotrimeric CST complex. The telomerase RNP is positioned at the chromosome terminus by an unknown recruitment factor (X) during S phase. TEN1 is displaced. POT1a (Pa) contacts STN1 (S) and CTC1 (C) to promote a telomere extendable state. POT1a also stimulates telomerase enzymatic properties. TEN1 represses telomerase activity and thus may help to terminate telomerase action. Telomerase is removed and replaced by POLα for C-strand fill-in and terminal DNA processing. The telomere is then converted into an un-extendable state.

Altogether, our data suggest a model in which POT1a facilitates telomere maintenance in two ways: by promoting the switch from the un-extendable to the extendable state and by stimulating telomerase enzyme activity (Fig. 7). In S phase, telomerase holoenzyme is recruited to the G-overhang through an unknown mechanism. The enzyme associates with CTC1 and STN1 through contacts with POT1a, and POT1a stimulates G-strand synthesis. One attractive hypothesis is that mobilization of POT1a to the chromosome terminus triggers the exchange of the telomerase negative regulator TEN1 from STN1 as part of the switch to the telomerase extendable state. Although our *in vitro* data indicate that STN1 has a higher affinity for TEN1 than POT1a OB1, additional contacts by other regions of POT1a or between POT1a and CTC1 may stabilize its interaction with STN1. Furthermore, shifting telomerase-CST interactions are likely to be governed by cell cycle specific posttranslational modifications such as those described for yeast Est1 and CST, as well as human TPP1 [19], [20], [37]. Once the G-strand is extended, telomerase action is terminated, perhaps with the assistance of TEN1. This clears the way for conventional replication machinery and processing enzymes to complete telomere replication and return the telomere to its fully protected un-extendable state. Although additional studies are needed to precisely delineate the telomere-telomerase interface and its control during telomere replication, our findings underscore the highly dynamic nature of telomerase-telomere transactions and suggest that modulation of telomerase enzyme activity at the chromosome terminus contributes to the bimodal switch in telomere states.

## Materials and Methods

### Plant materials

Plants were housed in growth chambers with a 16 hr photoperiod at 22°C. *stn1-1*, *ctc1-1*, *tert*, *pot1a-1* and *ten1-3* mutants were used for crosses and genotyped as described [38], [39], [41], [59]. *pot1a ter2* crosses were generated from homozygous parents. F1 progeny was planted for selection by genotyping. F3 seedlings were used for ChIP assays and pTRAP. *In vivo e*xperiments examining telomerase activity, protein interactions, or gene expression were either performed in juvenile seedlings or flowers, which both exhibit high levels of telomerase activity. For telomere length analysis, wild type controls were segregated from heterozygous parents to ensure that changes reflect mutations in the target genes and not stochastic variation [73].

### Chromatin immunoprecipitation

Approximately 4–6 grams of *Arabidopsis* seven day-old seedlings were used for each genotype. The protocol was adapted from [74] with minor changes. Sonication was performed on ice after crosslinking and nuclei extraction using (Fisher Scientific) with 4 cycles of 15 sec on and 1 min off per sample at 40% amplification. Immunoprecipitation (IP) was performed using rabbit anti-TERT antibody and Protein-A agarose/salmon sperm DNA beads (Millipore). Eluted DNA was subjected to Southern dot blotting using a telomeric [32P] 5′ end-labeled oligonucleotide probe. Stripping and rDNA hybridization performed as previously described using a combination of 5S (5′-TTGCAGAATCCCGTGAACCATCGAGT-3′) and 18S (5′-TGGAGCCTGCGGCTTAATTTGACTCA-3′) rDNA oligo-probes [59]. Quantification was performed on at least three independent biological replicates using Quantity One software (Bio-Rad).

### *E. coli* protein purification

Constructs for *E. coli* expression of TEN1 and POT1a OB1 were cloned in pET28a vector (Novagen). The POT1a OB1 domain was cloned from the POT1a start codon to residue 158. Four amino acids (SISS) were added to the C-terminus to increase protein solubility. Affinity column purification was achieved using Ni-NTA agarose resin (Qiagen) from BL21 DE3 lysates. Protein was eluted in imidazole buffer and dialyzed overnight. POT1a OB1 was further purified using a Sephadex G-75 (GE Healthcare) size exclusion column. TEN1 and POT1a OB1 protein fractions were analyzed for homogeneity on coomassie stained SDS-PAGE gels and verified by mass spectrometry. Proteins were expressed in rabbit reticulocyte lysate (RRL) (Promega) as indicated according to the manufacturer's instructions with [S35] Met (Perkin-Elmer) to label the protein expressed from pCITE4a, and in some cases pET28a.

### Protein interaction assays

POT1a, STN1, TEN1, and CTC1ΔN cDNA were cloned into pET28a (T7-tag fusion) and pCITE4a vectors (Novagen). Details for POT1a OB1, OB1+2, and C-terminus constructs are previously described [53]. Co-IP with the RRL-expressed proteins was performed as described [75]. Competition assays were performed by incubating *E. coli* TEN1 protein with RRL-expressed STN1, and various amounts of *E. coli* POT1a OB1 or BSA. Equal loading for STN1 was achieved by evenly dividing a single master mix of RRL-expressed protein among the samples. Pull downs were performed by IP of TEN1 using purified TEN1 antibody [41] and protein-A agarose beads (Pierce). Complexes were washed 10× with buffer W300 [75] and eluted by boiling for 5 min in SDS loading dye. Samples were resolved on 12% SDS-PAGE gels followed by coomassie staining and then dried for analysis by autoradiography.

### Protein immunoprecipitation

Extracts from ∼5 grams of wild type and *pot1a* seedling tissue were prepared as previously described [76] and pre-cleared using protein-A agarose beads (Pierce) with gentle rocking at 4°C for 1 h. IP was performed by adding 15 µg of affinity purified TERT, STN1, TEN1 or anti-GFP (Abcam) antibody (or pre-immune sera) overnight with gentle rocking at 4°C. Anti-rabbit STN1 antibody was raised from *E. coli* expressed and purified MBP-STN1 antigen. Protein-A agarose beads were added the following day for 2 hrs followed by 5× washes with buffer W300 [75], and 2× washes with buffer TMG [75]. IP samples were left in a final 50∶50 slurry in buffer TMG.

### Telomere and telomerase assays

DNA from whole plants was extracted as described [77]. TRF analysis was performed using 50 µg of DNA digested with *Tru1*I (Fermentas) and hybridized with a [P32] 5′ end–labeled (TTTAGGG)_4_ probe [76]. Blots were developed using a Pharos FX Plus Molecular Imager (Bio-Rad) and data were analyzed with Quantity One software (Bio-Rad). Primer extension telomere repeat amplification (PETRA) was performed as described [63]. 2 µg of DNA was used per reaction for telomere extension, followed by PCR amplification. PETRA products were separated on an agarose gel and subjected to Southern blotting using the same telomeric probe mentioned above.

Protein for Telomere Repeat Amplification Protocol (TRAP) assays were extracted from 5 day-old seedlings and reactions were conducted as described [76]. TRAP assays on STN1, TEN1, CTC1-CFP, or TERT IP samples were performed by using 1 µl of the final IP slurry. The telomerase processivity TRAP (TP-TRAP) protocol was adapted from [61] and performed as previously described [41]. Briefly, TP-TRAP entails telomerase extension of a substrate primer followed by the first round of PCR with a 1RPgg primer to incorporate a unique sequence tag on telomerase products. A primer complementary to the tagged region (2RP) is added for the second PCR step followed by 33× cycles of PCR. Relative telomerase activity was measured by Quantitative TRAP (qTRAP) via SYBR Geen (Bio-RAD) qPCR after primer extension as discussed [78].

### Western blotting

Fifty micrograms of wild type, *stn1*, and *pot1a* extracts were used for input samples. IP samples were boiled for 5 min in SDS loading dye. Samples were run on a 12% SDS-PAGE gel followed by protein gel blotting. Proteins were transferred overnight at 4°C onto a polyvinylidene difluoride (PVDF) membrane, followed by 2 hrs of blocking using 6% non-fat dried milk dissolved in 1× TBS-T (50 mM Tris, 150 mM NaCl, 0.1% Tween-20). Rabbit anti-STN1 antibody was diluted 1∶5000 in TBS-T and incubated with the protein blot for 4 hrs followed by 3× washes with TBS-T. Secondary anti-rabbit horseradish peroxidase was diluted 1∶7500 in TBS-T and incubated with the protein blot for 2 hrs, followed by 3× washes with TBS-T. Final detection was performed using an ECL prime protein blotting kit (GE Healthcare). Western blotting was performed as described for CTC1-CFP and POT1a [59] and TEN1 [41].

### Quantitative RT-PCR

RNA was extracted from 5 day-old seedlings (Omega Bio-tek) followed by DNase I digestion (Zymogen) for 30 min at room temperature. RNA was phenol: chloroform extracted followed by EtOH precipitation. 1 µg of RNA was reverse transcribed (Quanta Supermix), then diluted 1∶4 using thousand-fold diluted yeast tRNAs. 1 µl of cDNA was used for qRT-PCR using CFX Connect Real-Time System (Bio-Rad) in triplicate. Quantification is from three biological replicates and normalized to wild type for each gene expression.

## Supporting Information

Figure S1 Quantitative Real Time PCR (qRT-PCR) of telomere gene transcripts in wild type and *pot1a* plants. Results are shown for three independent biological replicates. Error bars represent standard error of the mean.
(TIF)

Figure S2 Telomerase processivity TRAP (TP-TRAP) assay. A five telomere repeat sequence attached to the typical TRAP substrate primer was used as a synthetic telomerase product control. Gel shows results from conventional TRAP reaction performed with a complementary telomere repeat reverse primer (left lane) and TP-TRAP reaction performed with the two unique reverse primers (right lane). Product size is slightly higher due to incorporation of the unique sequence tag.
(TIF)

Figure S3 TP-TRAP analysis of *ctc1* mutants. Results for flower extracts of wild type, *pot1a*, and *ctc1* sibling segregants of the genotypes indicated. Homozygous null plants display wild type product profiles.
(TIF)

Figure S4 Morphological and telomere length analysis of *ctc1 tert* mutants. (A) Morphological analysis of wild type, *ctc1, tert*, and *ctc1 tert* segregants. (B) PETRA analysis for the indicated genotypes was performed using a primer corresponding to the left arm of chromosome 1 (1L).
(TIF)

Figure S5 POT1a OB1 binds STN1 *in vitro*. RRL-expressed T7-tagged POT1a, OB1, OB1+OB2, and C-terminus was used to IP [^35^S] labeled STN1. (I) Input and (B) bound are shown.
(TIF)

Figure S6 CTC1 associates with active telomerase *in vivo*. (A) *In vivo* pull down of CTC1-CFP from transgenic Arabidopsis followed by western blot to detect GFP-CTC1 and POT1a. CTC1 and POT1a were detected by anti-GFP or anti-POT1a antibodies. Arrow indicates POT1a band and asterisk denotes a non-specific contaminant. Negative control is untransformed wild type tissue. Loading was monitored by Ponceau stain of IP samples. (B) Results of TRAP following IP of CTC1-CFP from transgenic Arabidopsis. Negative control was performed in untransformed wild type tissue.
(TIF)
