# Supplementary figures and images for: POT1a and Components of CST Engage Telomerase and Regulate Its Activity in Arabidopsis
Source: PLoS Genet. 2014 Oct 16;10(10):e1004738. doi: 10.1371/journal.pgen.1004738 (PMC4199523; doi:10.1371/journal.pgen.1004738)

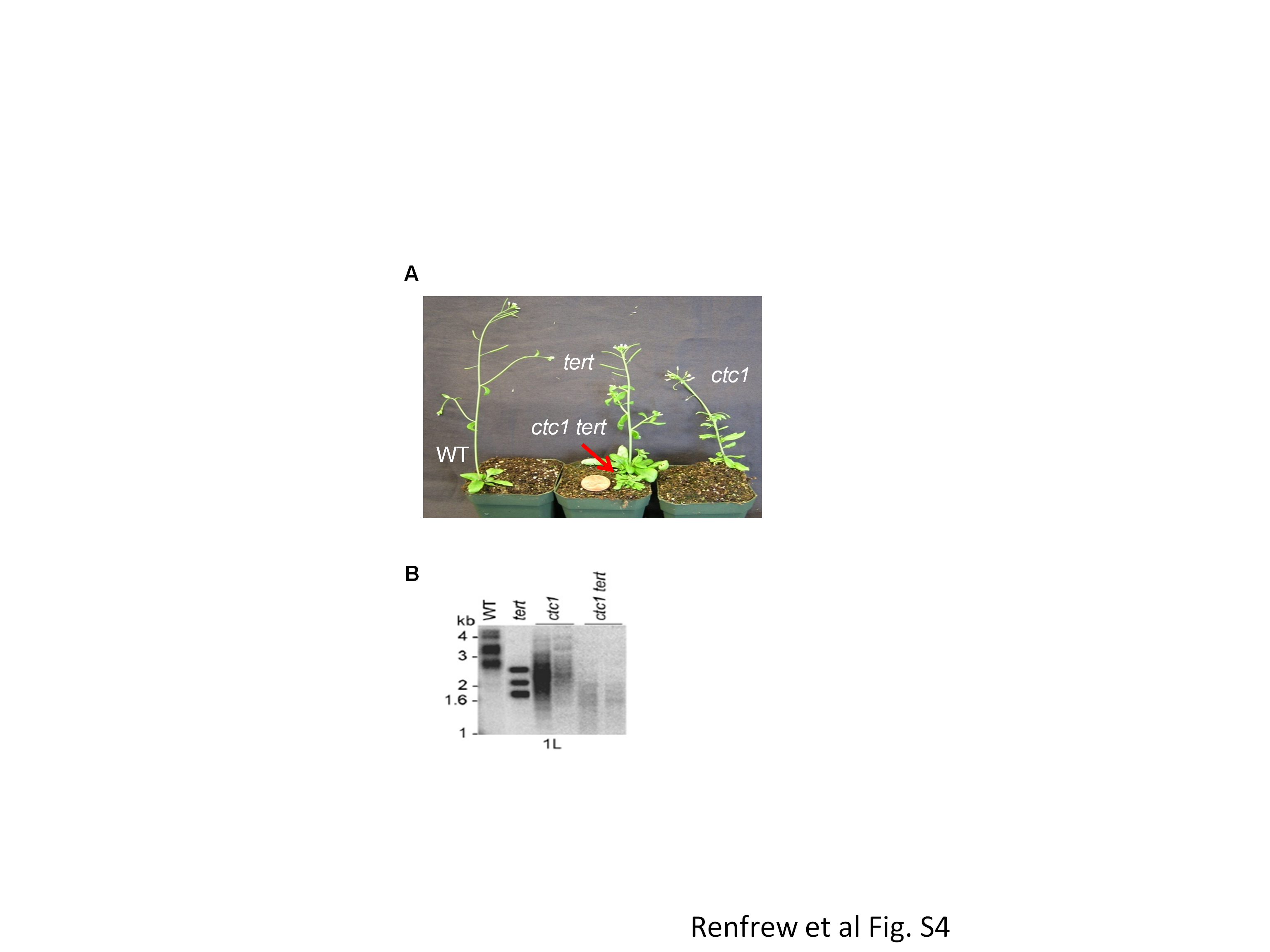

Supplement: Figure S4 — Morphological and telomere length analysis of ctc1 tert mutants. (A) Morphological analysis of wild type, ctc1, tert, and ctc1 tert segregants. (B) PETRA analysis for the indicated genotypes was performed using a primer corresponding to the left arm of chromosome 1 (1L). (TIF) [file pgen.1004738.s004.tif]

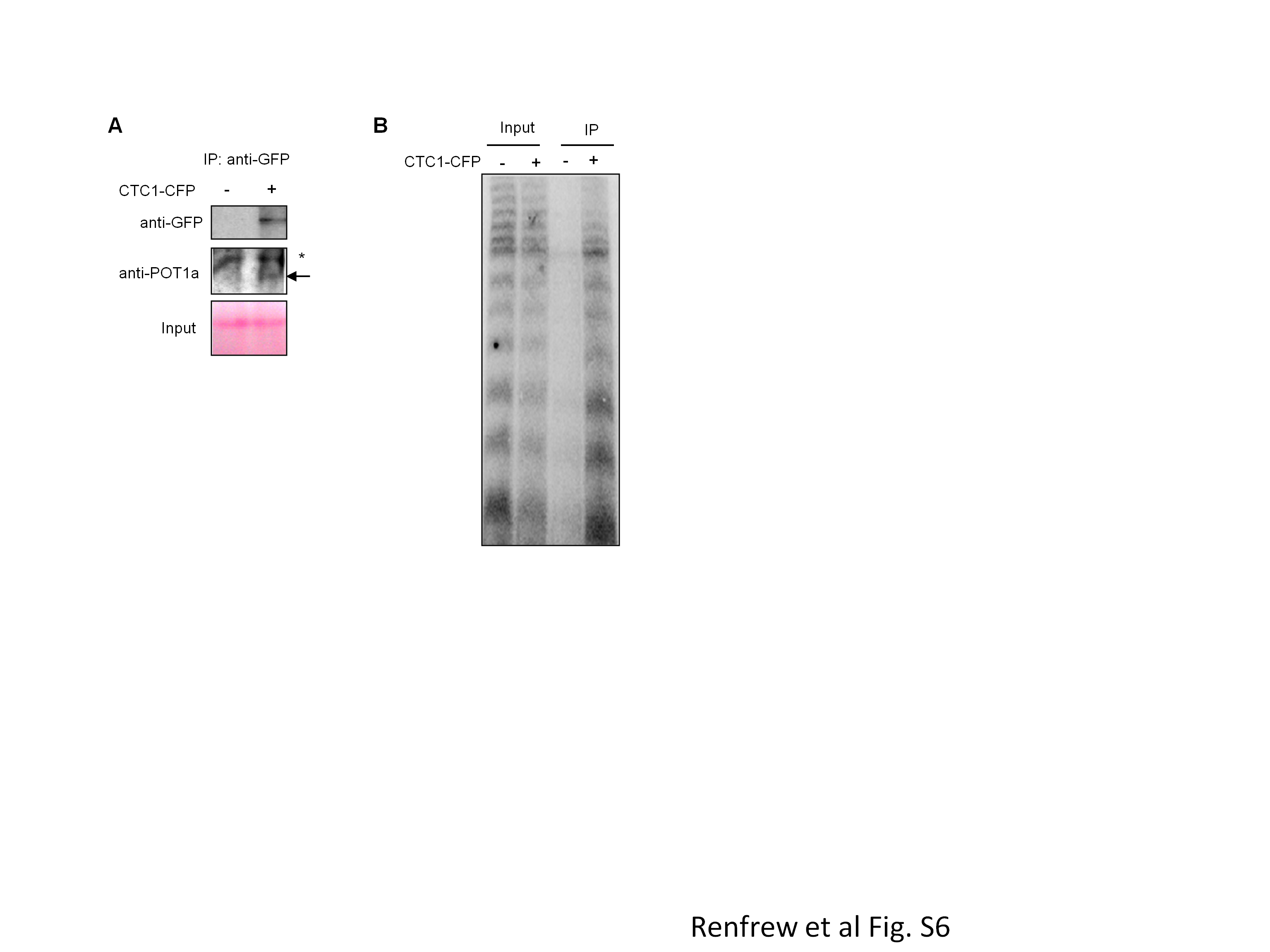

Supplement: Figure S6 — CTC1 associates with active telomerase in vivo. (A) In vivo pull down of CTC1-CFP from transgenic Arabidopsis followed by western blot to detect GFP-CTC1 and POT1a. CTC1 and POT1a were detected by anti-GFP or anti-POT1a antibodies. Arrow indicates POT1a band and asterisk denotes a non-specific contaminant. Negative control is untransformed wild type tissue. Loading was monitored by Ponceau stain of IP samples. (B) Results of TRAP following IP of CTC1-CFP from transgenic Arabidopsis. Negative control was performed in untransformed wild type tissue. (TIF) [file pgen.1004738.s006.tif]
